# 烟草烟雾通过ROS/Sirt3/SOD2通路诱导NSCLC细胞吉非替尼耐药

**DOI:** 10.3779/j.issn.1009-3419.2023.106.05

**Published:** 2023-04-20

**Authors:** ZI Yawan, LIAO Ke, CHEN Hong

**Affiliations:** ^1^400016 重庆，重庆医科大学附属第一医院呼吸与危重科（訾亚婉，廖科，陈虹）; ^1^Department of Pulmonary and Critical Care Medicine; ^2^重庆市眼科学重点实验室（訾亚婉）; ^2^Chongqing Key Laboratory of Ophthalmology, The First Affiliated Hospital of Chongqing Medical University, Chongqing 400016, China

**Keywords:** 肺肿瘤, 烟草烟雾, ROS, Sirt3/SOD2, 吉非替尼耐药, Lung neoplasms, Cigarette smoke, ROS, Sirt3/SOD2, Gefitinib resistance

## Abstract

**背景与目的** 表皮生长因子受体（epidermal growth factor receptor, EGFR）基因突变是非小细胞肺癌（non-small cell lung cancer, NSCLC）最常见的驱动基因突变。为延长患者生存时间，NSCLC EGFR酪氨酸激酶抑制剂（tyrosine kinase inhibitors, TKIs）耐药是目前急需解决的重大难题。本研究主要探究烟草烟雾（cigarette smoke, CS）诱导NSCLC发生吉非替尼耐药的机制。**方法** 体外培养PC-9、A549细胞，分别经1 µmol/L吉非替尼处理4 h、10%CS萃取物（CS extract, CSE）处理48 h。Western blot检测沉默蛋白3（Sirtuin 3, Sirt3）、超氧化物歧化酶2（superoxide dismutase 2, SOD2）蛋白表达，使用DCFH-DA探针检测细胞内活性氧（reactive oxygen species, ROS）水平，CCK-8试剂盒检测细胞活性，EdU检测细胞增殖能力。Sirt3过表达质粒（OV-Sirt3）转染于PC-9和A549细胞中、N-乙酰半胱氨酸乙酯（N-acetylcysteine, NAC）作用于细胞后分别经1 µmol/L吉非替尼处理4 h和10%CSE处理48 h。Western blot检测Sirt3、SOD2蛋白表达，DCFH-DA探针检测细胞中的ROS水平，CCK-8检测细胞活性。**结果** CSE均可促使PC-9、A549细胞对吉非替尼的半数抑制浓度（50% inhibitory concentration, IC_50_）提高（P<0.01），并且增强PC-9和A549细胞的增殖能力，提示CS可诱导NSCLC吉非替尼耐药；ROS参与CSE诱导的吉非替尼耐药（P<0.05）；CSE诱导Sirt3、SOD2低表达（P<0.01），且Sirt3/SOD2与肺癌患者不良预后有关（P<0.05）；OV-Sirt3的PC-9、A549细胞可逆转CSE诱导的吉非替尼耐药（P<0.05）且显著降低ROS生成；NAC可逆转CSE诱导的PC-9、A549细胞吉非替尼耐药（P<0.05）。**结论** ROS/Sirt3/SOD2通路参与了CS诱导的NSCLC吉非替尼耐药。

## Abstract (en)

**Background and objective** Epidermal growth factor receptor (EGFR) gene mutations are the most common driver mutations in non-small cell lung cancer (NSCLC). To prolong the survival of the patients, EGFR tyrosine kinase inhibitors (TKIs) resistance in NSCLC is a major challenge that needs to be addressed urgently, and this study focuses on investigating the mechanism of cigarette smoke (CS) induced Gefitinib resistance in NSCLC. **Methods** PC-9 and A549 cells were cultured in vitro and treated with 1 µmol/L Gefitinib for 4 h and 10% cigarette smoke extract (CSE) for 48 h. Western blot was used to detect Sirtuin 3 (Sirt3) and superoxide dismutase 2 (SOD2) protein expressions; DCFH-DA probe was used to detect intracellular reactive oxygen species (ROS); CCK-8 kit was used to detect cell activity, and EdU was used to detect cell proliferation ability. Sirt3 overexpression plasmid (OV-Sirt3) was transfected in PC-9 and A549 cells and treated with 1 µmol/L Gefitinib for 4 h and 10% CSE for 48 h after N-acetylcysteine (NAC) action. The expressions of Sirt3 and SOD2 were detected by Western blot; the ROS level in the cells was detected by DCFH-DA probe, and the cell activity was detected by CCK-8. **Results** CSE induced an increase in the 50% inhibitory concentration (IC_50_) of both PC-9 and A549 cells to Gefitinib (P<0.01) and enhanced the proliferation of PC-9 and A549 cells, suggesting that CS induced Gefitinib resistance in NSCLC. ROS was involved in CSE-induced Gefitinib resistance (P<0.05). CSE induced low expressions of Sirt3 and SOD2 (P<0.01), and Sirt3/SOD2 was associated with poor prognosis in lung cancer patients (P<0.05). OV-Sirt3 in PC-9 and A549 cells reversed CSE-induced Gefitinib resistance (P<0.05) and significantly reduced ROS production. NAC reversed CSE-induced Gefitinib resistance in PC-9 and A549 cells (P<0.05). **Conclusion** The ROS/Sirt3/SOD2 pathway is involved in CS-induced Gefitinib resistance in NSCLC.

**【Competing interests】**The authors declare that they have no competing interests.

至今，肺癌仍是世界上导致癌症相关死亡的主要原因^[1]^，其中非小细胞肺癌（non-small cell lung cancer, NSCLC）是肺癌发生率最高的类型^[2]^。既往研究^[3⇓-5]^发现表皮生长因子受体（epidermal growth factor receptor, EGFR）基因特异性突变的NSCLC患者与酪氨酸激酶抑制剂（tyrosine kinase inhibitors, TKIs）吉非替尼临床反应相关，吉非替尼可参与阻断NSCLC细胞增殖和侵袭的信号通路，目前吉非替尼被推荐作为EGFR突变NSCLC患者的一线化疗药物^[6]^。虽然EGFR-TKIs给患者带来了显著的临床获益，但随着疗程延长、基因突变等因素极易引起获得性耐药的发生，70%-80%的NSCLC患者对吉非替尼耐药，并导致疾病进展^[7]^。因此，攻克吉非替尼耐药性这一大难题是提高NSCLC生存率的主要挑战，且对于找到有效的干预措施至关重要。

耐药细胞中活性氧（reactive oxygen species, ROS）和抗氧化酶系统失衡是导致耐药性产生的机制之一^[8]^。具有强大的线粒体去乙酰化酶活性的沉默调节蛋白3（Sirtuin 3, Sirt3）^[9]^，可参与控制ROS以调节线粒体正常功能运转^[10,11]^，抑制细胞凋亡，是肿瘤发生的关键调节因子。实验^[9]^证明Sirt3通过调控线粒体生理、抵抗应激诱导的超氧化水平和基因组不稳定性而发挥肿瘤抑制作用，那么清除ROS的Sirt3也可能参与调控肿瘤耐药，然而此方面研究甚少。烟草烟雾（cigarette smoke, CS）可促进肺癌、乳腺癌、结直肠癌、食管癌等肿瘤细胞的增殖、侵袭和迁移，因而吸烟可增加癌症发生和进展的风险，其中与肺癌相关性最高^[12]^。吸烟是肺癌发生和发展的主要危险因素，也是EGFR突变的影响因素，且影响EGFR-TKIs的疗效^[13]^。但目前尚未有研究报道Sirt3在吸烟诱导耐药机制中的作用。本研究主要在于探究CS导致EGFR-TKIs吉非替尼耐药的作用机制，为临床解决NSCLC吉非替尼耐药提供理论思路。

## 1 材料与方法

### 1.1 细胞培养与材料

EGFR突变型的吉非替尼敏感的人肺腺癌细胞株（PC-9）和EGFR野生型的人肺腺癌细胞株（A549）均购于上海赛百慷生物公司。PC-9和A549细胞使用含有10%胎牛血清（fetal bovine serum, FBS）的DMEM高糖培养基，置于37 ^o^C、含有5%CO_2_的细胞恒温培养箱中，培养箱湿度为70%-80%。FBS（P30-3306）购于德国PAN-Biotech公司。DMEM（11965092）高糖培养基购于美国Gibco公司。转染试剂Lipofectamine 2000（11668019）购于美国ThermoFisher科技有限公司。Sirt3过表达质粒由上海汉恒生物公司构建。Sirt3兔单抗、超氧化物歧化酶2（superoxide dismutase 2, SOD2）兔单抗均购于江苏亲科（Affinity）生物研究中心有限公司，β-actin兔单抗（1:1,000）抗体购于英国Abcam公司，辣根酶标记山羊抗兔IgG二抗购于美国亚科因（Abbkine）生物科技公司。吉非替尼（Gefitinib, ZD1839）药物、N-乙酰半胱氨酸乙酯（N-acetylcysteine, NAC, HY-B0215）均购于美国MCE公司。CCK-8细胞增殖或毒性检测试剂盒购于日本同仁化学研究所。ROS检测试剂盒、EdU细胞增殖检测试剂盒（DAB法）均购于上海碧云天生物科技研究所。

### 1.2 CS萃取物（CS extract, CSE）的提取

CSE的制备是根据以下参考文献中的方法进行改良而来^[14,15]^。将一支红双喜牌香烟（含有13 mg焦油、1.3 mg尼古丁）烟雾溶于1 mL DMEM高糖基础培养基中，每支香烟燃烧至少5 min。完全燃烧5支香烟共制得5 mL CSE-高糖DMEM溶液，并将pH调整为7.40，后用0.22 µm孔径的无菌滤器过滤除菌。设定此浓度为100%CSE，在后续实验中用DMEM培养基稀释至所需的浓度，于-80 ^o^C冰箱储存备用。

### 1.3 Western blot实验

将各组处理之后的细胞，用适宜的预冷RIPA裂解液和PMSF混合液裂解细胞30 min后，提取总蛋白质。使用BCA蛋白浓度试剂盒检测各组的蛋白浓度，再加入5×蛋白上样缓冲液于沸水中煮10 min进行蛋白变性。上样于10%SDS聚丙烯酰胺凝胶，电泳仪中电泳分离蛋白，PVDF转膜、5%脱脂奶粉封闭2 h。后放于稀释的目的蛋白一抗中4 ^o^C孵育过夜，抗体稀释浓度：Sirt3兔单抗（1:1,000）、SOD2兔单抗（1:1,000），后于室温中二抗孵育2 h，曝光显影。以上实验均至少重复3次，使用Image J软件进行条带灰度值分析。

### 1.4 细胞活性的检测（CCK-8）

将对数生长期且生长状态良好的PC-9和A549细胞分组并每孔5,000个细胞接种于96孔板中，待其贴壁。使用高糖DMEM培养基将提取的CSE稀释为10%。（1）吉非替尼组：使用高糖DMEM培养基将吉非替尼稀释为不同浓度（1 µmol/L、5 µmol/L、10 µmol/L、50 µmol/L、100 µmol/L和500 µmol/L），并依次加入96孔板中培养24 h；（2）吉非替尼+10%CSE组：1 µmol/L吉非替尼对PC-9和A549细胞预处理4 h，然后与10%CSE共孵育48 h；（3）吉非替尼+10%CSE组+OV-Sirt3组：1 µmol/L吉非替尼对成功转染Sirt3过表达质粒的PC-9和A549细胞预处理4 h，然后与10%CSE共孵育48 h；（4）吉非替尼+10%CSE组+NAC组：先对PC-9和A549细胞于5 µmol/L NAC预处理4 h，然后于1 µmol/L吉非替尼处理4 h，最终与10%CSE共孵育48 h。去除旧培养液加入100 µL无血清培养基，每孔加入10 µL CCK-8溶液，设置没有细胞但有实验组等量的细胞培养液、CCK-8溶液的孔作为空白对照组。在细胞培养箱中孵育1 h-1.5 h后，使用酶标仪检测450 nm的吸光度。以上实验均重复3次。

### 1.5 EdU DAB法检测细胞增殖

取对数生长期的PC-9和A549细胞种于6孔板中，且每组细胞数量相同，培养箱中贴壁后加10%CSE或吉非替尼，继续培养48 h后检测细胞增殖。将37 ^o^C预热的10 µmol/L的EdU工作液加入孔板中孵育2 h后，用4%多聚甲醛固定15 min。PBS清洗3次后，0.3% Triton X-100通透15 min。PBS清洗3次后，内源性过氧化物酶封闭液室温孵育20 min。之后根据试剂盒说明书配制Click反应液，室温避光孵育30 min。PBS清洗3次后，根据说明书配制Streptavidin-HRP工作液，室温孵育30 min。使用DAB显色后，于普通光学显微镜下观察并拍照记录。

### 1.6 过表达质粒转染细胞

Lipofectamine 2000转染试剂是一种用于将核酸（DNA和RNA）转染到真核细胞中的专利配方。分组为：转染Sirt3过表达质粒的PC-9组（OV-Sirt3 PC-9）、转染空载质粒的PC-9组（OV-NC PC-9）、转染Sirt3过表达质粒的A549组（OV-Sirt3 A549）、转染空载质粒的A549组（OV-NC A549）。转染前一天，尽可能使细胞的汇合度在转染时达到70%-80%。对于每个转染样品，如下制备寡聚物-Lipofectamine 2000复合物：（1）在不含血清的50 µL Opti-MEM减血清培养基中稀释20 pmol过表达质粒；（2）使用前轻轻混合Lipofectamine 2000，然后在50 µL Opti-MEM减血清培养基中稀释，轻轻混合，并在室温下孵育5 min；（3）将稀释的寡聚物与稀释的Lipofectamine 2000轻轻混合，并在室温下孵育20 min。将寡聚体-Lipofectamine 2000复合物加入到每个含有细胞和培养基的孔中，4 h-6 h后可更换培养基，再将细胞在37 ^o^C的CO_2_培养箱中培养24 h-96 h。Western blot验证转染效果。

### 1.7 免疫荧光（immunofluorescence, IF）实验

将种于无菌载玻片的经相应处理后的PC-9和A549细胞用PBS清洗2次，使用多聚甲醛固定15 min，在加入抗体孵育前，需用0.3%Triton X-100进行细胞通透，以保证抗体能够到达抗原部位。使用正常山羊血清封闭30 min后，根据Sirt3（1:200）、SOD2（1:200）稀释目的蛋白抗体，进行一抗4 ^o^C孵育过夜，后予以荧光二抗室温避光孵育2 h，最后DAPI染细胞核及封片和荧光显微镜检查。

### 1.8 ROS检测

通过DCFH-DA荧光探针进行ROS检测。相应处理后的PC-9和A549细胞经PBS清洗后，加入1 mL 1:1,000用无血清培养液稀释的DCFH-DA，于细胞培养箱内避光孵育30 min。用PBS缓冲液或无血清DMEM基础培养基清洗细胞3次，每次至少1 min，以充分去除未进入细胞的DCFH-DA。应用488 nm激发波长、525 nm发射波长于荧光显微镜下检查拍照。以上实验至少重复3次，使用Image J软件分析荧光光度值。

### 1.9 统计学分析

使用SPSS 26.0软件进行统计学分析，符合正态分布的计量结果均使用均数±标准差（Mean±SD）表示。应用GraphPad Prism 9.3.1软件计算半数抑制浓度（50% inhibitory concentration, IC_50_）值。两独立组之间的比较，若两组满足正态分布且方差齐，则选用t检验；多独立组之间的分析，若组间呈正态分布则选用单因素方差分析（one-way ANOVA）。若组间不服从正态分布，则选用秩和检验。P<0.05表示差异具有统计学意义。

## 2 结果

### 2.1 CS引起NSCLC发生吉非替尼耐药性

为了探讨CSE暴露是否会诱导NSCLC吉非替尼耐药，我们检测了PC-9（EGFR外显子19突变型）细胞和A549（EGFR野生型）细胞不同组的IC_50_。细胞活力曲线显示：（1）在PC-9细胞中，10%CSE+吉非替尼组的IC_50_（143.70±0.14）µmol/L明显高于对照组IC_50_（8.87±0.64）µmol/L，10%CSE刺激后PC-9对吉非替尼的耐药性是对照组的16.20倍，且差异具有统计学意义（P<0.01）（图1A）；（2）在A549细胞中，10%CSE+吉非替尼组的IC_50_（93.14±1.02）µmol/L明显高于对照组（9.19±1.12）µmol/L，10%CSE刺激后A549对吉非替尼的耐药性是对照组的10.13倍，且差异具有统计学意义（P<0.01）（图1B）。以上结果显示，CSE可诱导EGFR突变的PC-9细胞和EGFR野生型的A549细胞发生吉非替尼耐药。

**图1:**
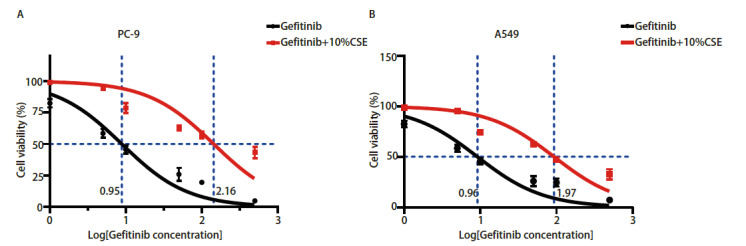
烟草烟雾引起非小细胞肺癌发生吉非替尼耐药性。 CCK-8法检测不同剂量吉非替尼和10%CSE处理PC-9（A）和A549（B）细胞48 h后的细胞活力。

### 2.2 ROS参与CS诱导的NSCLC吉非替尼耐药

已有研究^[16]^表示ROS参与了CSE诱导的NSCLC细胞系EGFR-TKIs耐药。为了验证ROS在CSE诱导的吉非替尼耐药中的作用，我们将1 µmol/L吉非替尼对PC-9和A549细胞预处理4 h，然后与10%CSE共孵育48 h，通过DCFH-DA探针检测了CSE处理的PC-9和A549细胞中的ROS变化。结果显示在PC-9和A549细胞中，10%CSE处理后ROS水平均显著上升，且吉非替尼不能抑制（图2A）。接下来，我们通过EdU DAB法检测了10%CSE刺激PC-9和A549细胞后的增殖能力变化，以此探究CSE对NSCLC细胞系增殖活力的影响。实验结果显示，10%CSE分别刺激PC-9和A549细胞后，其增殖能力明显增加（图2B）。综上所得，CSE通过促进NSCLC细胞ROS增加而导致其增殖活力增强，其中EGFR突变型PC-9细胞增殖能力更为明显。总之，ROS参与了CS诱导的NSCLC细胞吉非替尼耐药。

**图 2:**
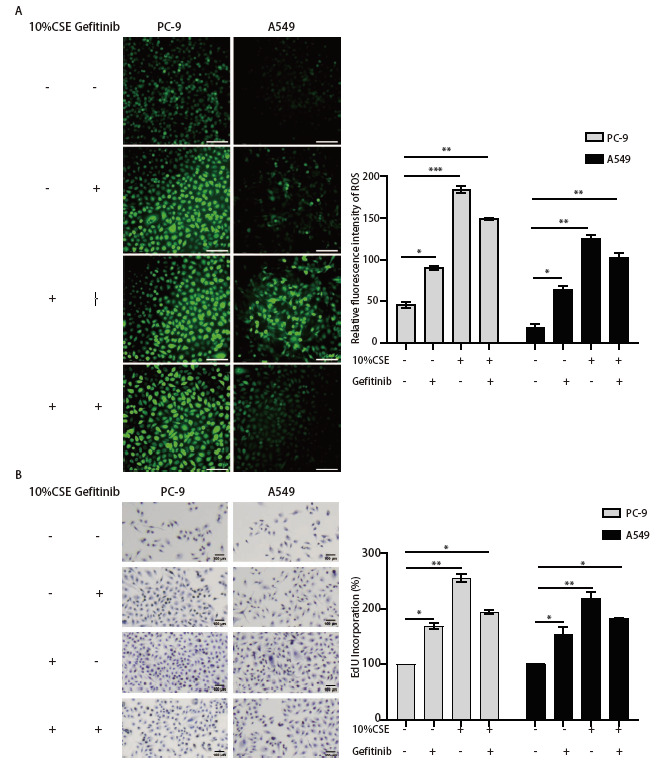
活性氧参与烟草烟雾诱导的NSCLC吉非替尼耐药。 A：DCHF-DA探针检测10%CSE和吉非替尼处理PC-9、A549细胞的活性氧水平（比例尺=100 µm），以及经Image J测量后的统计学分析；B：EdU DAB法检测了10%CSE、Gefitinib按照组别分别处理PC-9和A549细胞后的增殖能力（比例尺=100 µm）以及Image J测量后的统计学分析。*P<0.05; **P<0.01; ***P<0.001。

### 2.3 Sirt3/SOD2与肺癌患者不良预后相关

通过检索GEPIA数据库发现，相较于癌旁组织，无论是肺腺癌还是肺鳞癌，Sirt3均表达较低。此外，我们通过The Human Protein Atlas数据库的生存分析，以无进展生存期（progression-free survival, PFS）为筛选终点，基于2,437例具有吸烟史的接受放化疗的肺癌患者，发现低表达Sirt3患者的PFS明显缩短（P<0.05）。综上，Sirt3在NSCLC中低表达，而低水平Sirt3预示着较差的化疗反应，因此我们选择Sirt3作为探究CS诱导NSCLC EGFR-TKIs吉非替尼耐药机制的关键蛋白。

Sirt3通过去乙酰化SOD2上的两个关键氨基酸残基促进其抗氧化的活性，则SOD2是Sirt3的靶基因，激活Sirt3/SOD2通路可明显降低ROS和提升氧化应激抗性的能力^[17]^。通过检索GEPIA数据库发现Sirt3与SOD2之间具有明显的正相关性（P=3e^-06^<0.01，r=0.24，图3A），两者具有蛋白互作关系。经Western blot实验发现10%CSE处理后在PC-9和A549细胞中Sirt3、SOD2均表达降低（图3B），通过IF实验验证其表达情况与Western blot结果一致，且Sirt3、SOD2均定位于细胞质（图4），提示CSE刺激NSCLC细胞后Sirt3及SOD2均表达降低。

**图 3:**
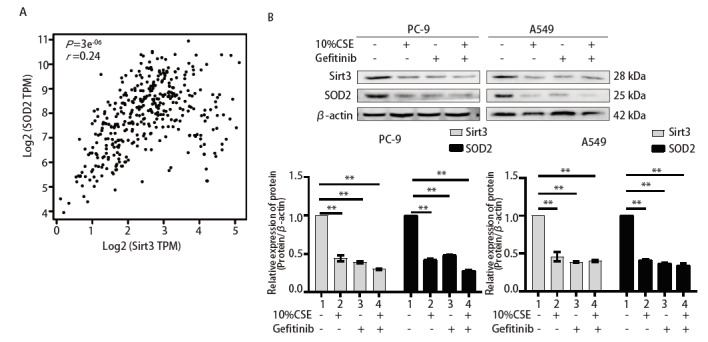
Sirt3/SOD2与肺癌患者不良预后有关。 A：GEPIA数据库检索Sirt3与SOD2之间表达相关性；B：Western blot检测Sirt3、SOD2的表达情况，以及Image J灰度值分析后的统计学。**P<0.01。

**图 4:**
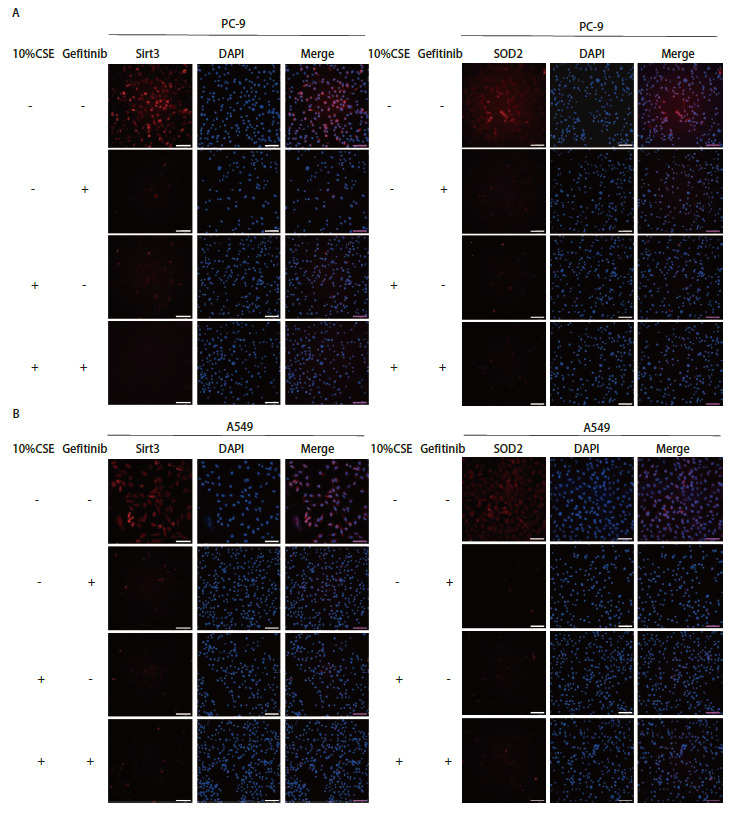
免疫荧光检测Sirt3和SOD2在PC-9（A）和A549（B）细胞中的表达和定位（比例尺=100 µm）

### 2.4 Sirt3/SOD2参与CS诱导的NSCLC吉非替尼耐药

为了验证Sirt3在CS诱导NSCLC细胞吉非替尼耐药中的作用，我们构建了过表达Sirt3的PC-9细胞（OV-Sirt3 PC-9）和A549细胞（OV-Sirt3 A549），并通过Western blot验证了其构建成功，且SOD2随着Sirt3过表达而上调，说明SOD2是Sirt3的下游靶蛋白（图5A）。经CCK-8验证：（1）PC-9细胞中10%CSE+OV-Sirt3+吉非替尼组的IC_50_（6.98±1.24）µmol/L明显低于10%CSE+吉非替尼组（106.86±1.04）µmol/L（图5B），可见OV-Sirt3后PC-9细胞对吉非替尼的敏感性是10%CSE的15.31倍，差异具有明显统计学意义（P<0.01）；（2）A549细胞中10%CSE+OV-Sirt3+吉非替尼组的IC_50_（11.13±1.15）µmol/L明显低于10%CSE+吉非替尼组（83.24±1.02）µmol/L（图5B），可见OV-Sirt3后的A549细胞对吉非替尼的敏感性是10%CSE的7.48倍，且差异具有明显统计学意义（P<0.01）。以上结果说明过表达Sirt3可逆转CSE诱导的NSCLC吉非替尼耐药。

**图 5:**
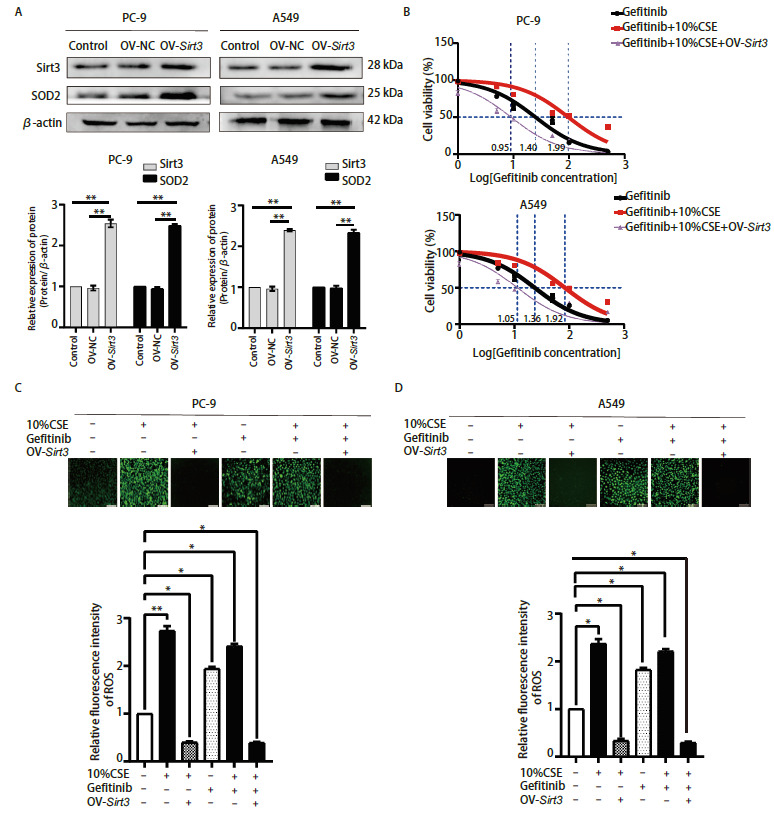
Sirt3/SOD2参与烟草烟雾诱导的NSCLC吉非替尼耐药。 A：Western blot验证OV-Sirt3质粒和转染PC-9和A549细胞后Sirt3和SOD2的表达情况，以及Image J灰度值分析后的统计学；B：不同剂量吉非替尼、10%CSE分别处理OV-Sirt3 PC-9细胞和OV-Sirt3 A549细胞后的细胞活性。C、D：吉非替尼、10%CSE分别处理OV-Sirt3 PC-9（C）和OV-Sirt3 A549（D）细胞后的ROS水平变化，以及Image J荧光光度分析后的统计学（比例尺=100 µm）。*P<0.05；**P<0.01。

接下来为了验证Sirt3是否抑制CSE诱导NSCLC产生ROS，我们检测了OV-Sirt3 PC-9细胞、OV-Sirt3 A549细胞内的ROS水平。结果显示，过表达Sirt3后明显抑制了CSE诱导PC-9（图5C）和A549（图5D）细胞产生的ROS。综上得出，激活Sirt3/SOD2信号通路可明显抑制CSE诱导NSCLC细胞系产生的ROS，同时增强了EGFR突变型肺腺癌细胞对吉非替尼的敏感性，说明其可逆转NSCLC吉非替尼耐药性。因此针对Sirt3的靶向药有可能成为扭转耐药细胞生存的重要治疗方向。

### 2.5 抑制ROS可逆转CS诱导的NSCLC细胞吉非替尼耐药

我们又进一步地验证了ROS是否影响Sirt3/SOD2的作用，以及两者在CSE诱导PC-9、A549细胞吉非替尼耐药的作用。使用ROS抑制剂即5 µmol/L NAC预处理4 h后经10%CSE或1 µmol/L吉非替尼处理48 h，我们发现NAC作用后10%CSE、吉非替尼处理PC-9和A549细胞后的ROS显著降低（图6A）。IC_50_结果显示：（1）PC-9细胞中10%CSE+NAC+吉非替尼组的IC_50_（5.74±1.06）µmol/L明显低于10%CSE+吉非替尼组（122.85±1.51）µmol/L（图6B），可见NAC预处理后PC-9细胞对吉非替尼的敏感性是10%CSE的21.40倍，差异具有明显统计学意义（P<0.01）；（2）A549细胞中10%CSE+NAC+吉非替尼组的IC_50_（9.81±0.85）µmol/L同样明显低于10%CSE+吉非替尼组的（103.50±0.66）µmol/L（图6B），可见预处理后A549细胞对吉非替尼的敏感性是10%CSE的10.55倍，且差异具有明显统计学意义（P<0.01），提示NAC可逆转CSE诱导的NSCLC吉非替尼耐药。

**图6:**
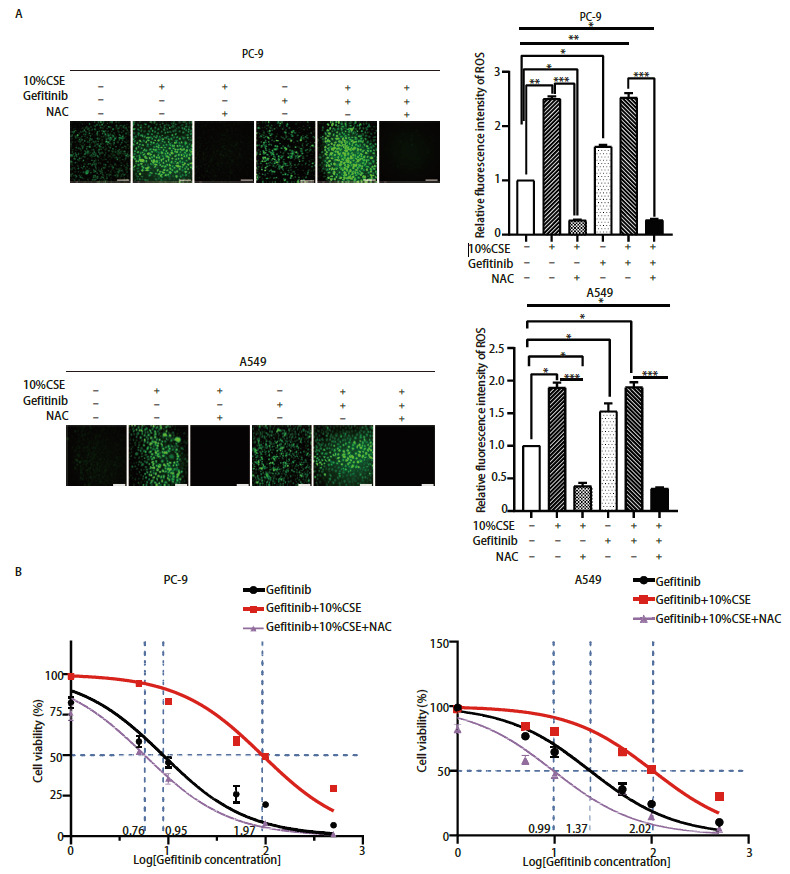
抑制ROS可逆转烟草烟雾诱导的NSCLC细胞吉非替尼耐药。 A：5 µmol/L NAC预处理PC-9和A549细胞后经吉非替尼、10%CSE刺激后的ROS水平变化（比例尺=100 µm)，以及Image J荧光光度分析后的统计学；B：NAC预处理PC-9和A549细胞4 h后经吉非替尼、10%CSE刺激48 h后的细胞活力。*P<0.05；**P<0.01；***P<0.001。

经Western blot发现NAC处理后Sirt3、SOD2表达均明显上调（图7），表示抑制ROS可激活Sirt3/SOD2表达。以上结果显示，无论是EGFR突变型还是EGFR野生型NSCLC细胞，抑制ROS可显著逆转CSE诱导的NSCLC吉非替尼耐药，同时ROS/Sirt3/SOD2通路贯穿于此发生机制中，起到重要的调节作用。

**图 7:**
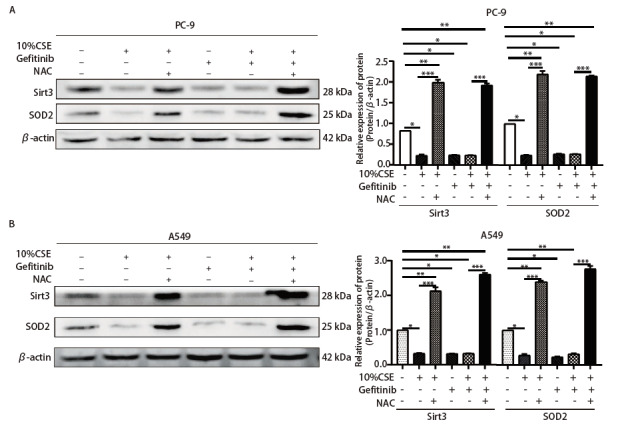
Western blot检测NAC预处理PC-9（A）和A549（B）细胞4 h后经吉非替尼、10%CSE处理48 h后Sirt3、SOD2的表达情况，以及Image J灰度值分析后的统计学。 *P<0.05；**P<0.01；***P<0.001。

## 3 讨论

NSCLC最常见的驱动基因突变是EGFR基因突变，发生率为30%-40%，随着肺癌的精准治疗，EGFR-TKIs已经成为EGFR基因突变局部晚期或转移NSCLC患者的标准治疗方案，但大多数患者会在用药后一段时间发生耐药，之后EGFR-TKIs的疗效甚微，不足以延长患者生存时间。因此，EGFR-TKIs耐药的针对性机制研究对突破耐药问题极为重要。

研究^[18⇓⇓-21]^显示，在肺癌诊断后继续吸烟会降低50%的生存率，部分原因是吸烟降低了癌症治疗的效果。同时吸烟史对EGFR-TKIs的疗效有明显影响，对于目前具有吸烟史的EGFR突变的耐药患者，即使厄洛替尼的剂量从最适低剂量至300 mg，不仅肿瘤的生长不能被有效抑制，而且总生存期（overall survival, OS）及PFS也没有延长^[22,23]^。除此之外，吸烟也是导致吉非替尼治疗的NSCLC患者的进展后生存期（post-progressive survival, PPS）缩短的独立危险因素^[24]^。我们的研究发现，无论是EGFR突变型还是EGFR野生型的NSCLC，CS均可诱导其IC_50_明显延长，对吉非替尼的耐药性增加数倍，这可能是吸烟导致吉非替尼治疗效果大幅度降低的主要原因。

现有报道^[25]^显示烟草中主要成分尼古丁影响健康的机制有氧化应激、DNA损伤及损伤血管内皮等。和癌症相关的一些不利因素（如烟草、辐射等）都能通过产生ROS，与细胞相互作用激活各种转录因子，导致肿瘤细胞存活、增殖和侵袭等，同时还与化疗抵抗和防止细胞死亡有关^[26,27]^。肿瘤耐药可能的机制理论有氧化应激、DNA损伤修复、抗凋亡和免疫逃逸等^[28]^。并且有研究^[8,29]^表明，肿瘤细胞内显著增加的ROS水平与各种放化疗耐药性发生密切相关，吉非替尼治疗过程中细胞ROS增加导致线粒体功能障碍，以此产生耐药性。我们发现CS均可以导致NSCLC细胞ROS水平增加，且EGFR突变型增加较为明显；抑制ROS后NSCLC细胞的IC_50_明显降低，显示其对吉非替尼的敏感性增加，这些结果均表示CS引起的ROS升高是诱导NSCLC耐药性产生的一个主要机制。肿瘤细胞内部活动如增殖、侵袭和迁移，癌基因突变，肿瘤微环境变化等情况均严格依赖ROS的积累^[27]^，因此肿瘤细胞的生存发展和ROS变化密切相关。本研究发现，CS促使NSCLC细胞ROS增加，同时强化了肿瘤细胞增殖活力，说明CS诱导NSCLC细胞氧化应激而致EGFR-TKIs耐药及促进增殖能力。

Sirt3是位于细胞线粒体内的参与调节线粒体氧化途径的酶及相关转录因子的主要去乙酰化酶^[30]^，其重要功能是消除ROS参与维持细胞稳态。多项研究已经表明，在NSCLC^[31]^、结肠癌^[32]^和肝癌^[33]^的治疗中，Sirt3的高表达可以明显提高肿瘤患者化疗效果，因此Sirt3可以作为提高肿瘤患者化疗敏感性的重要靶点。既往有报道^[32]^，敲低Sirt3基因后结肠癌细胞内的ROS水平增加，并且相关抗氧化系统的酶表达显著降低，重要的是结肠癌细胞对化疗药物的敏感性明显提高，表示Sirt3可参与化疗药物耐药的机制形成。SOD2是Sirt3主要的下游蛋白，Sirt3可通过SOD2去乙酰化增强细胞抵抗氧化应激的能力，提高细胞存活^[34]^。我们的研究表示，CS可引起NSCLC细胞Sirt3和SOD2表达降低，两者的表达与ROS水平呈负相关，并且过表达Sirt3后ROS明显降低且NSCLC细胞系对吉非替尼的敏感性增加，我们的研究验证了CS与肺癌吉非替尼耐药之间存在着密切联系，并且可通过调控Sirt3/SOD2通路活性影响ROS来改善耐药问题，为解决耐药问题提供新方向，但还需要进一步了解Sirt3/SOD2是如何通过改变ROS来促进药物治疗对肿瘤微环境的反应，也是探究耐药机制的主要方向。

综上，通过本研究可得出，CS可通过抑制Sirt3/SOD2通路促使NSCLC细胞产生ROS，诱导其发生吉非替尼耐药。戒烟可有效降低ROS水平，可显著缓解EGFR突变的NSCLC患者的肿瘤进展^[23]^。因此，对于化疗和/或靶向治疗的肺癌患者，戒烟是增强疗效的有效措施，也是提高生存率的重要举措。
